# Jackets of Sodium Cilicate in the Treatment of Spinal Disease

**Published:** 1886-04-20

**Authors:** S. T. De La Mater

**Affiliations:** Fayetteville, N. Y.


### JACKETS OF SODIUM SILICATE IN THE TREATMENT OF SPINAL DISEASE.

BY S. T. DE LA MATER, M.D., OF FAYETTVILLE, N. Y.

I have had such satisfactory results from the use of sodium silicate for jackets in treating spinal disease, curvatures, etc., and the comfort to the patient has been so great that I wish to say a few words in its favor to the readers of your valuable journal. Perhaps some one may not know of its use in this direction and may be anxiously wishing for a substitute for the heavy plaster of Paris, let me say to them you have a most desirable one in the silicate, and after once using, it will meet your warmest approval. Very frequently the potassium silicate is sold you by the druggist for the sodium ; do not accept it. You will be disappointed in it every time you use it. Insist on having the sodium. Remember its color is light amber when pure and the consistency of syrup, while the potassium is pale white.

Its superiority over plaster is in its being more cleanly, lighter, requiring only a few layers to obtain the desired support, and hardens quickly. It requires a little more dexterity it its application, but after a little practice you will be able to handle it easily and quickly. In using it for the jacket in spinal disease, I first fit the under shirt snugly to the person—a very necessary point; then cover with one layer of cotton wadding; over this I usually roll one layer of plaster; this gives you a good foundation for the silicate, then cutting my strips of cheese cloth one yard long, 2½ inches wide, I roll them on, moistening them with the silicate as I proceed. Afterward, with the hand or brush, entirely covering the surface with the silicate. Three or four layers of the cloth will be sufficient for a jacket on a child, and this will not weigh more than six or eight ounces, while with the plaster it would be several pounds.

In one hour you will find the surface hard and firm, ready to be trimmed, and the patients will tell you how very comfortable they feel, scarcely realizing they have any dressing on. When carefully fitted and trimmed there is no discomfort, rather a feeling of security, which is always grateful in spinal diseases, and there being so little thickness to the dressing, there will be no necessity for changing the clothing; a very satisfactory matter in many cases.

To more fully illustrate the advantages of this dressing, let me speak of an interesting case now under observation.

A little boy now 4 years of age, while being drawn in a cart, was thrown violently backwards upon his shoulder and back. This occurred over one year ago. The usual symptoms of Pott's disease developed, and the family doctor, a fair surgeon, applied a plaster jacket. Not having had much experience, he made the first one too short, reaching only to the short ribs. This, after wearing two weeks with great discomfort, was taken off and another one, larger and fitting perfectly, applied. This, after wearing a short time, had to be removed on account of its weight. At no time could the little fellow stand on his feet but a few hours, and not then without the support of a chair or something to lean against. The parents had about given up all hope of ever benefitting him with a jacket.

When brought to my office, three months ago, he was walking with his hands upon his knees, not being able to straighten up even with support but for a few minutes. A very prominent knuckle had formed in the dorsal region, and the chest wall upon the left side was badly depressed, and the heart showed the effect of compression by the ribs. He was rapidly losing flesh; was pale and countenance pinched. His rapid and oppressed breathing was painful to hear; appetite gone; constipated; and sleep was fitful, broken by dreams and startings. I applied a silicate jacket at once, using for suspenion a swing which was designed by Dr. Squire, of New York, and of which I will speak more fully later. I used the same methods of applying the silicate spoken of, and after trimming and fitting it to his body, set him on his feet and told him to go. He did so at once, running around the room, laughing and playing, and from that day to this he has been like other children, going to school and church—plays out of doors at all times, and the mother tells me he eats and sleeps like any child in perfect health. His red cheeks and plump limbs tell the best story.

I always put my patients with spinal disease immediately upon Hydroleine, and the good effect is seen at once in their rapid increase in flesh, and children begin to grow in height as well. This little fellow, just spoken of, has grown in three months two and a half inches.

Now, I wish to say a few words about the swing spoken of; not only its use for applying the jacket, but its daily use for extension afterward. No good result can be expected unless the after treatment by daily use of some mode of extension can be employed, and how will you obtain this with children? The self-extension swing I have spoken of is par excellence for this purpose, and no matter how poor the patient may be, they can have one at a cost not to exceed one dollar.

I have had the swing, with all the parts in position as it appears ready for use, photographed so that any person can make one from seeing the photograph. This photograph will be sent to any one who desires it for twenty-five cents. Those having had much experience in the treatment of spinal curvatures know how difficult it is to get their patients to come daily for extension, and the cost of a tripod, too, is large. Now, for one dollar, every patient can have an extension swing, and in their own homes, where it can be easily hung in a doorway and taken down when not in use.—*New England Medical Monthly.*

